# Suppression of Heterogeneous Nuclear Ribonucleoprotein C Inhibit Hepatocellular Carcinoma Proliferation, Migration, and Invasion *via* Ras/MAPK Signaling Pathway

**DOI:** 10.3389/fonc.2021.659676

**Published:** 2021-04-16

**Authors:** Jiejun Hu, Dong Cai, Zhibo Zhao, Guo-Chao Zhong, Jianping Gong

**Affiliations:** Department of Hepatobiliary Surgery, The Second Affiliated Hospital of Chongqing Medical University, Chongqing, China

**Keywords:** HCC, HNRNPC, alternative splicing, Ras, MAPK

## Abstract

Hepatocellular carcinoma (HCC), the most common malignant tumor, has high fatality and recurrence rates. Accumulating evidence shows that heterogeneous nuclear ribonucleoprotein C (HNRNPC), which is mainly involved in RNA splicing, export, and translation, promotes progression and metastasis of multiple tumor types; however, the effects of HNRNPC in HCC are unknown. In the present study, high levels of HNRNPC were detected in tumor tissues compared with para-tumor tissues by immunohistochemical and western blot assays. Furthermore, Cox proportional hazards regression models, the Kaplan–Meier method, and clinicopathologic features analysis showed that HNRNPC was not only an independent prognostic factor for both overall and disease-free survival in HCC but also a predictor of large tumor size and advanced tumor stage. Functional experiments revealed that silencing of HNRNPC not only led to arrest of more HCC cells at G0/G1 phase to inhibit their proliferation, but also suppressed EMT process to block their invasion, and migration *in vitro*; this was related to the Ras/MAPK signaling pathway. In addition, blocking of HCC cell proliferation regulated by HNRNPC silencing was observed *in vivo*. Finally, rescue tests showed that after recovery of Ras/MAPK signaling pathway activity by treatment with Ras agonists, the proliferation, migration, and invasion suppression of Huh-7 and Hep 3B cell lines caused by HNRNPC knockdown was partially reversed. Taken together, these results indicate that HNRNPC knockdown inhibits HCC cell proliferation, migration and invasion, in part *via* the Ras/MAPK signaling pathway. Thus, HNRNPC may have an important role in the progression of HCC and represents a promising biomarker for evaluation of prognosis and a potential therapeutic target in HCC patients.

## Introduction

Hepatocellular carcinoma (HCC) is the fifth most common cancer and the fourth leading cause of cancer-associated deaths worldwide, with a gradually increasing global burden (1, 2). For patients with early-stage tumors, liver resection and transplantation are the main treatment modes (3–5), whereas for patients with advanced-stage tumors, multimodal treatment is increasingly popular; this includes immunological therapy, molecular targeted treatments, microwave ablation, and transarterial chemoembolization (6–11). Although an increasing number of therapies are used to treat HCC, the prognosis of HCC patients remains poor (12–14), with a 5-year overall survival rate of less than 20% (15–17), primarily owing to the difficulty of early-stage diagnosis (18). For these reasons, there is a need to explore novel biomarkers that could be used as targets for HCC therapy and for predicting the prognosis of HCC patients.

Alternative splicing enables the generation of vast protein diversity by cutting out the introns and connecting the exons of heterogeneous nuclear RNAs (19). Increasing numbers of reports indicate that alternative splicing is intimately related to tumor occurrence, progression, and therapeutic resistance (20–23). A known RNA-binding protein, heterogeneous nuclear ribonucleoprotein C (HNRNPC) plays an important part in RNA splicing (24, 25), stability (26, 27), and expression (28). HNRNPC has been identified as an oncogene, enhancing deterioration in multiple tumor types, including gastric cancer (29), breast cancer (27), esophageal squamous cell carcinoma (26), and oral squamous cell carcinoma (30). According to previous reports, HNRNPC primarily regulates the biological activity of the IFNβ signaling pathway (27), p53 gene (31), and AKT signaling pathway (32), which are associated with tumor proliferation, invasion, and metastasis. Bioinformatic analysis has shown that HNRNPC, as a gene correlated with N6-methyladenosine RNA methylation, predicts poor prognosis of patients with glioblastoma multiforme (33), lung adenocarcinoma (34), and head and neck squamous cell carcinoma (35). Thus, HNRNPC is considered to be a tumor-related gene. Nevertheless, its impact on patient prognosis and the mechanism by which it regulates the biological characteristics of tumor cells in HCC remain unknown.

The mitogen-activated protein kinase (MAPK) cascade consists of serine/threonine kinases, which mainly participate in the transduction of cellular signals (36, 37). There are four important protein kinases, Ras, Raf, MEK, and Erk, in the MAPK signaling pathway (38). In response to signals from epidermal growth factor receptor, insulin like growth factor-1 receptor (IGF-1R), and calmodulin, the GDP combined with Ras is replaced by GTP. Then, the activated Ras cascade phosphorylates its downstream targets Raf, MEK, and Erk to regulate cell proliferation, differentiation, and cycling (39–41). Additionally, Ras/MAPK signaling pathway promoting epithelial-mesenchymal transition (EMT) to facilitate tumor migration and invasion have been reported in recent years (42–44). Abnormal activation of the MAPK pathway is involved in the progression of many malignancies, including gastric cancer (45), cervical carcinoma (46), and HCC (47). Hence, targeting Ras/Raf/MEk/Erk is regarded as a potential approach to treating HCC (36), esophageal carcinoma (48), acute lymphoblastic leukemia (49), and melanoma (50).

In the present study, we used western blotting, immunohistochemistry (IHC), and bioinformatics analysis to demonstrate that HNRNPC had significantly elevated expression in HCC tumor tissues compared with para-tumor tissues. In addition, high levels of HNRNPC protein were related to larger tumor size, advanced TNM stage, and poor prognosis. Subsequently, functional experiments revealed that silencing of HNRNPC expression could inhibit HCC cells’ growth by G0/G1 arrest, in part *via* the Ras/MAPK signaling pathway. Meanwhile, *in vitro* tests, we also demonstrated that the inhibition of Ras/MAPK signaling pathway caused by HNRNPC suppression could negatively regulate EMT progress to hinder migration and invasion of HCC cells. In vivo, xenograft assays showed that HNRNPC knockdown could block the proliferation of HCC cells, with a statistically significant effect. Therefore, HNRNPC may be a promising biomarker for evaluating prognosis of HCC patients, as well as a potential therapeutic target.

## Materials and Methods

### Sample Collection

In order to detect differences in HNRNPC protein levels between tumor tissues and para-tumor tissues, we collected 12 pairs of fresh tissues (tumor and matched para-tumor tissue) from patients diagnosed with HCC by a pathologist after undergoing hepatectomy between Dec 2018 and Jan 2019 in the Department of Hepatobiliary Surgery of the Second Affiliated Hospital of Chongqing Medical University. For analysis of prognosis and clinical information, our study enrolled 147 available cases from Jul 2014 to Nov 2016 from the same department who had not received chemotherapy, radiofrequency ablation, or molecular targeted therapy before liver resection. These 147 cases included 128 male and 19 female patients, with an average age of 53.67 years. Specific patient information is given in **Table 1**. The selection criteria were as follows. Inclusion criteria: HCC confirmed by biopsy after surgery; exclusion criteria: patients with other cancers, distant metastasis, or grade C Child–Pugh liver function. A total of 147 tumor tissues and 82 available corresponding para-tumor tissues were used to construct tissue microarrays (TMAs). This research was approved by the Ethics Committee at the Second Affiliated Hospital of Chongqing Medical University ((2017)36), and informed consent was obtained from all patients.

**Table 1:** Relationships of HNRNPC protein levels with clinicopathologic features in 147 HCC patients.

Clinicopathologic features	All patients (n=147)	High HNRNPC (n=80)	Low HNRNPC (n=67)	P-value
Gender				
Male	128	70 (54.7%)	58 (45.3%)	1.000
Female	19	10 (52.6%)	9 (47.4%)	
Age (M ± SD)	53.67 ± 10.21	53.94 ± 9.95	53.48 ± 10.49	0.784
<55 y	82	46 (56.1%)	36 (43.9%)	0.739
≥55 y	65	34 (52.3%)	31 (47.7%)	
HBV infection				
negative	25	16 (64.0%)	9 (36.0%)	0.379
positive	122	64 (52.5%)	58 (47.5%)	
Liver cirrhosis				
absent	55	33 (60.0%)	22 (40.0%)	0.310
present	92	47 (51.1%)	45 (48.9%)	
AFP				
<400 ng/ml	89	45 (50.6%)	44 (49.4%)	0.310
≥400 ng/ml	58	35 (60.3%)	23 (39.7%)	
Child–Pugh				
A	120	63 (52.5%)	57 (47.5%)	0.395
B	27	17 (63.0%)	10 (37.0%)	
Tumor number				
1	126	65 (51.6%)	61 (48.4%)	0.103
2–3	21	15 (71.4%)	6 (28.6%)	
Tumor size (M ± SD)	5.36 ± 3.09	5.99 ± 3.41	4.61 ± 2.48	**0.007**
< 5 cm	72	32 (44.4%)	40 (55.6%)	**0.021**
≥5 cm	75	48 (64.0%)	27 (36.0%)	
TNM stage				
I	88	41 (46.6%)	47 (53.4%)	**0.028**
II–III	59	39 (66.1%)	20 (33.9%)	
Tumor differentiation				
I–II	42	25 (59.5%)	17 (40.5%)	0.468
III–IV	105	55 (52.4%)	50 (47.6%)	

HNRNPC, heterogeneous nuclear ribonucleoprotein C; HBV, hepatitis B virus; AFP, alpha fetoprotein. The bold values present statistical significance.

### Follow-Up

Follow-up was carried out by phone, and alpha fetoprotein (AFP), liver function, and chest radiography were obtained at least every 2 months in the first 6 months after surgery, every 3 months for 6 months to 3 years following surgery, and every 6 months for 3–5 years after surgery. If necessary, magnetic resonance imaging or computed tomography was also used. Overall survival was defined as the time from liver resection to death or last follow-up; disease-free survival was defined as the time from liver resection to first recurrence or last follow-up.

### IHC

Fresh tissues were immobilized with 4% formalin for 48 h at 37°C and subsequently washed with cool phosphate-buffered saline (PBS) three times for 5 min each time. Tissue samples were dehydrated using ethanol, embedded in paraffin, and then cut them into 4-μm-thick sections of 2 mm diameter to produce TMAs. The paraffin sections were dewaxed in an incubator at 65°C overnight, before being deparaffinized in xylene and rehydrated using a decreasing ethanol gradient. Sections were placed in ethylene diamine tetraacetic acid (cat.no. 0085; Beyotime) and heated in a microwave for 10 min for antigen repair. A 3% hydrogen peroxide was used to eliminate endogenous peroxidase at 37°C for 30 min. Sections were incubated with anti-HNRNPC (dilution 1:50, cat. no. ET1611-2; HUABIO), anti-ki67 (dilution 1:10000, cat. no. 27309; Proteintech), anti-CD34 (dilution 1:50, cat. no. ET1606-11; HUABIO), anti-Vimentin (dilution 1:100, cat. no. ET1610-39; HUABIO), and anti-E-Cadherin (dilution 1:50, cat. no. ET1607-75; HUABIO) antibodies at room temperature overnight, and then with secondary antibody (dilution: 1:1, cat.no. K5007; Dako) for 1 h at room temperature. Finally, sections were stained with an IHC kit (cat. no. K5007; Dako) according to the manufacturer’s protocol.

### IHC Scoring

The proportion of positive cells and staining strength were used to evaluate the expression of HNRNPC. Color intensity was ranked using four grades: no staining (score = 0), weak staining (score = 1), moderate staining (score = 2), and intense staining (score = 3); and the proportions of positive cells were divided into five classifications: < 5% (score = 0), 5–25% (score =1), 26–50% (score = 2), 51–75% (score = 3), and 76–100% (score = 4). These two values were multiplied to acquire the final score. All patients were divided into high and low HNRNPC expression groups according to the median score of 7. The IHC results were analyzed by two pathologists; uncertain results were judged by a third pathologist.

### Western Blot Analysis

We collected total protein from cells or tissues using RIPA buffer with protease and phosphatase inhibitors. After separation by sodium dodecyl polyacrylamide gel electrophoresis, proteins were transferred onto polyvinylidene fluoride membranes. Subsequently, the membranes were blocked with 5% skim milk at room temperature for 1 h, then incubated with primary antibody at 4°C overnight. On the second day, the membranes were incubated with secondary antibody for 1 h at 37°C. The antibodies used were as follows: HNRNPC (dilution 1: 2000, cat. no. ET1611-2; HUABIO), Ras (dilution 1:1000, cat. no. 3339T; CST), c-Raf (1:1000, cat. no. 9422T; CST), P-c-Raf (dilution 1:1000, cat. no. 9427T; CST), MEK1/2 (dilution 1:1000, cat. no. 8727T; CST), P-MEK1/2 (Ser217/221) (dilution 1:1000, cat. no. 9154T; CST), Erk1/2 (dilution 1:1000, cat. no. 4695T; CST), P-Erk1/2 (Thr202/Tyr204) (dilution 1:1000, cat. no. 4377T; CST), c-Myc (dilution 1:1000, cat. no. 5605T; CST), CDK4 (dilution 1:2000, cat. no. ET1612-1; HUABIO), cyclin E1 (dilution 1:1000, cat. no. ET1612-16; HUABIO), Vimentin (dilution 1:2000, cat. no. ET1610-39; HUABIO), E-Cadherin (dilution 1:1000, cat. no. ET1607-75; HUABIO), β-actin, (dilution 1:2000, cat. no. BM0627; Boster), goat anti-mouse (dilution 1:5000, cat. no. ZB-2305; ZSGB-BIO), and goat-anti-rabbit (dilution 1:5000, cat. no. ZB-2301; ZSGB-BIO). Finally, the membranes were imaged using a chemiluminescent horseradish peroxidase substrate (cat. no. WBKL S0100; Millipore). β-actin was used as the internal reference, and the western blot analysis was performed using Image Lab 6.0. The tests mentioned above were operated three times respectively.

### Quantitative Real-Time PCR (q-RT-PCR)

Total RNA was extracted from cells using an Animal Total RNA Isolation Kit (cat. no. RE-03011; Foregene) according to the manufacturer’s instructions. Total RNA was reverse transcribed to cDNA with an iScript cDNA synthesis kit (cat. no. 1708891; Bio-Rad), following the manufacturer’s reaction protocol: 5 min at 25°C 20 min at 46°C, 1 min at 95°C, and holding at 4°C. Quantitation of cDNA was performed using a SYBR Green PCR kit (cat. no. 208054; QIAGEN). Relative HNRNPC expression was calculated by the 2^-△△Cq^ method, with GAPDH as an internal reference. The primers were designed as follows: GAPDH forward, 5′-GGAGTCCACTGGCGTCTTCA-3′, reverse, 5′-GTCATGAGTCCTTC CACGATACC-3′; HNRNPC forward, 5′-GCAGAGCCAAAAGTGAACCG-3′, reverse, 5′-ACGTTTCGAGGGCACTACAG-3′. The reaction protocol was given by the manufacturer as follows: 2 min at 95°C, 5 sec at 95°C, and 10 sec at 60°C, repeated for 35 cycles. Each test was repeated dividedly in triplicate.

### Cell Lines and Culture

Huh-7 and Hep 3B HCC cell lines were purchased from the Chinese Academy of Sciences Stem Cell Bank (Shanghai, China) and identified with STR Profile. Both cell lines were cultured in DMEM (cat.no. SH30022.01; HyClone) or MEM (cat.no. SH30024.01; Hyclone) supplied with 10% fetal bovine serum (cat.no. 04-001-1ACS; BI) in an incubator containing 5% CO_2_ at 37°C.

### Cell Infection

Huh-7 and Hep 3B cells were cultured in six-well plates. When they reached 20–30% density, they were stably infected with HNRNPC lentivirus (Lv-HNRNPC) or a negative control lentivirus (Lv-NC) for 14 h (viral volume=MOI × cell number/viral titer; GeneChem, Shanghai, China). The lentiviral interference system was produced using a GV248 carrier loaded consecutively with the hU6 promoter, a multiple cloning site, the ubiquitin promoter, the EGFP gene, an IRES site, and the puromycin gene. A short hairpin RNA was designed to target the HNRNPC sequence (sh-HNRNPC): 5′-CTTCGTTCAGTATGTTAAT-3′; the negative sequence (sh-NC) was 5′-TTCTCCGAACGTGTCACGT-3′. After culture for 48 h, cells were filtrated with 2 µg/ml puromycin (cat. no. REVG1001; GeneChem, Shanghai, China) for 48 h; this was repeated twice. Infection and knockdown efficiency were detected by inverted fluorescence microscopy, and by western blotting and q-RT-PCR, respectively.

### Colony Formation Assay

Approximately 500 cells were seeded in each well of the six-well plates and cultured in an incubator with 5% CO_2_ at 37°C for 2 weeks. After washing with PBS, the cells were immobilized with 4% paraformaldehyde for 15 min at room temperature and then stained with 0.1% crystal violet solution (cat. no. G1062; Solarbio) for 30 min. Finally, the colony numbers were counted under a microscope. All experiments were operated independently three times.

### Cell Viability Analysis

Approximately 3000 (Huh-7) or 8000 (Hep 3B) cells were seeded in each well of a 96-well plate and cultured for 72 h. After being seeded in a 96-well plate, cell viability was measured using a CCK-8 kit (cat. no. ZP328-3; ZOMANBIO) according to the manufacturer’s instructions at 6, 24, 48 and 72h. Briefly, CCK-8 (10 µl) was added to each well, and the cells were incubated at 5% CO_2_ and 37°C. The absorbance (optical density) was detected by enzyme-linked immunosorbent array at a wavelength of 450 nm. Each test was performed dividedly in triplicate.

### Scratch Wound for Cell Migration Assay

When the cells had grown to a density of 90% in the six-well plates, we used a sterile 200-µl pipette tip to scratch a wound at the middle line of each well bottom. Subsequently, the suspended cells were removed by PBS, and the culture medium was replaced with serum-free medium. Cells were sequentially cultured for 48 h in an incubator containing 5% CO_2_ at 37°C. The wound width was recorded under a light microscope at 0, 24 and 48h. The experiments mentioned above were operated three times respectively.

### Cell Invasion Assay

To analyze the invasion ability of cells, a Corning BioCoat Matrigel Invasion Chamber (cat. no. 354480; Corning) was used according to the manufacturer’s instructions. Specifically, cells (5×10^4^/well) were cultured with serum-free medium in the upper chambers, while the lower chambers were loaded with DMEM or MEM supplied with 10% fetal bovine serum. After culture for 48 h, the cells were washed with PBS and fixed with 4% paraformaldehyde for 15 min at room temperature. Invading cells were captured with 0.1% crystal violet solution under a light microscope, and non-invading cells were eliminated with cotton swabs. Specifically, five fields in each chamber were randomly recorded, and average values were calculated. Each test was repeated independently in triplicate.

### Cell Cycle Analysis

Approximately 25×10^4^/well cells were seeded in six-well plates. Subsequently, the cells were collected in 1.5-ml EP tubes and then fixed with 70% ethanol at 4°C overnight. The next day, the cells were stained with propidium iodide with RNase A at 37°C for 30 min in the dark. Finally, the results were analyzed by flow cytometry. A total of 2×10^4^ cells were recorded for each sample. All experiments were performed three times respectively.

### Groups and Treatments

To verify the upstream and downstream relationships between HNRNPC and the MAPK signaling pathway, we used an agonist for Ras (ML-098) (cat. no. HY-19800; MCE). The specific treatments were as follows. To measure the efficiency of the activation of the MAPK signaling pathway by ML-098, the Lv-NC group was treated with dimethyl sulfoxide (DMSO) for 48 h, and the Lv-HNRNPC group was treated with ML-098 (20 nmol/l) and DMSO, for 48 h. CCK-8 tests, cell cycle analysis, scratch wound assay, and cell invasion tests were performed using the method described above. To detect the rescue efficiency of ML-098 for colony formation, we used ML-098 (20 nmol/l) to treat the Lv-HNRNPC group twice sequentially for 5 days each time. The Lv-NC group was treated with the same amount of DMSO as a control.

### *In Vivo* Assay for Tumor Growth

A total of 12 5-week-old male BALB/c nude mice were purchased from Beijing Huafukang Biotechnology and randomly divided into two groups (six per group). Lv-HNRNPC- and Lv-NC-group Huh-7 cells were suspended in cool PBS and subsequently injected into the backs of mice subcutaneously (2.5×10^6^/mice). The mice were fed at 23–25°C and 60% humidity with a 12-h light/dark cycle. Tumor size was measured with a caliper every 3 days; the formula for the size calculation was as follows: volume = (length × width^2^)/2 cm^3^. Twenty-seven days after implantation, the mice were killed by cervical dislocation and the tumors were removed. Finally, the tumor samples were fixed with 4% formalin for IHC analysis. All animal experiments were approved by the Ethics Committee of Chongqing Medical University.

### Bioinformatic Analysis

The expression of HNRNPC in HCC tissues was analyzed by UALCAN (ualcan.path.uab.edu), using RNA sequencing data downloaded from The Cancer Genome Atlas for 50 normal liver tissues and 371 primary HCC tissues. HNRNPC-correlated genes were also acquired from UALCAN; we selected 3517 significantly correlated genes (Pearson correlation coefficient ≥ 0.5) for Kyoto Encyclopedia of Genes and Genomes (KEGG) analysis by WebGestalt 2013 (www.webgestalt.org/2013).

### Statistical Analysis

IBM SPSS Statistics 23 was used for statistical analysis. Categorical data were analyzed by chi-square test, while continuous data between two groups were analyzed by t-test. Comparisons between multiple groups were performed by one-way analysis of variance (ANOVA), and the results were analyzed by LSD test and presented as mean ± standard deviation (SD). Univariate and multivariate Cox proportional hazards regression models were used to assess the risk factors associated with HCC. In addition, overall and disease-free survivals were computed by the Kaplan–Meier method and examined by log-rank test. Finally, a P-value less than 0.05 was considered to indicate a statistically significant difference.

## Results

### HNRNPC Is Upregulated in Tumor Tissues and Predicts Large Tumor Size, Advanced TNM Stage, and Poor Prognosis in HCC

**Figure 1:**
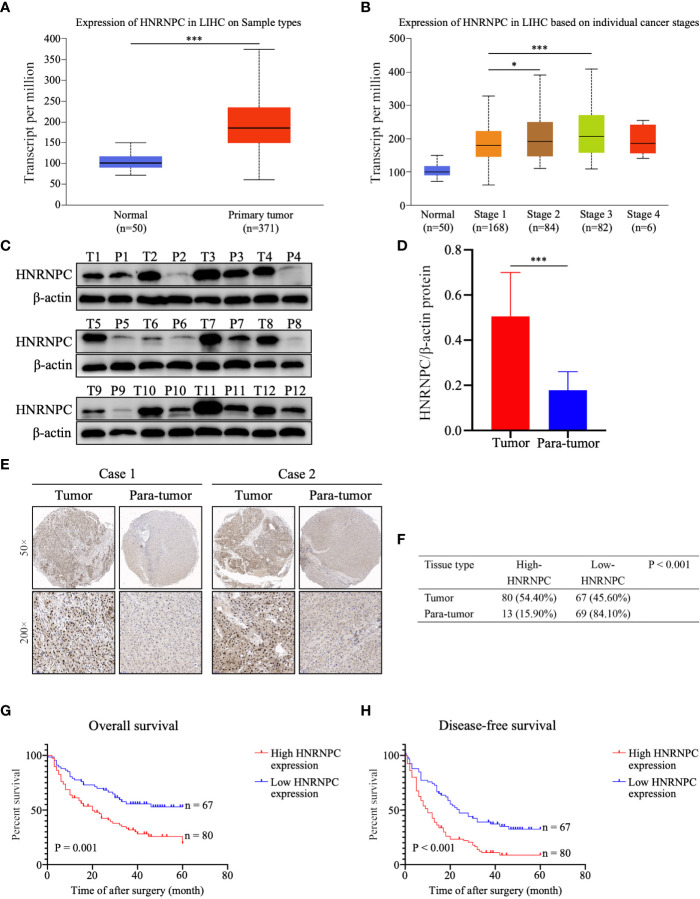
High HNRNPC expression was detected in HCC tumor tissues, and predicts a poor survival in HCC patients. **(A)** HNRNPC mRNA expression in HCC tissues and normal liver tissues acquired from UALCAN analysis. **(B)** UALCAN analysis showing HNRNPC mRNA expression at different cancer stages. **(C, D)** Western blot showing protein expression of HNRNPC in HCC tumor tissues and matched para-tumor tissues. **(E)** Classical cases showing HNRNPC protein expression in HCC tumor tissues and paired para-tumor tissues. **(F)** The χ2 test was used to assess HNRNPC protein expression in HCC tissues and adjacent tissues. **(G)** Kaplan–Meier analysis showing the correlation of HNRNPC level with overall survival of HCC patients. **(H)** Kaplan–Meier analysis showing the association between HNRNPC level and disease-free survival. The final results were presented as mean ± standard deviation (SD). *P < 0.05, ^***^P < 0.001.

Using the UALCAN database, we found that HNRNPC mRNA levels were higher in 371 primary HCC tissues than in 50 normal liver tissues (P < 0.001, **Figure 1A**); in addition, the expression of HNRNPC mRNA was elevated in stage 2 (P = 0.033, **Figure 1B**) and stage 3 (P < 0.001, **Figure 1B**) HCC patients compared with stage 1 HCC patients. To compare HNRNPC protein expression in tumor and para-tumor tissues, western blotting and IHC were performed. HNRNPC protein levels were obviously higher in tumor tissues than in para-tumor tissues, based on western blot analysis of 12 HCC tumor tissues and their matched para-tumor tissues (P< 0.001, **Figures 1C, D**). The IHC results showed high expression of HNRNPC protein, mainly located in nuclei, in 80 of 147 tumor tissues (54.40%) and in 13 (15.90%) of 82 paired para-tumor tissues (P<0.001, **Figures 1E, F**). Correlations of HNRNPC protein levels with clinicopathological features in HCC were analyzed by chi-square test; this demonstrated that HNRNPC protein levels were significantly associated with tumor size (P = 0.007) and tumor TNM stage (P=0.028) (**Table 1**). To explore the effects of HNRNPC protein levels on survival of HCC patients, Kaplan–Meier survival curves were constructed, showing that patients with high HNRNPC protein expression had poorer overall survival (P = 0.001, **Figure 1G**) and disease-free survival (P < 0.001, **Figure 1H**) than those with low HNRNPC protein expression. Univariate and multivariate analysis were used to evaluate the risk factors associated with HCC patients’ survival. According to our results, tumor size (P = 0.005, HR = 1.957), tumor TNM stage (P = 0.014, HR = 1.786), and high HNRNPC expression (P = 0.039, HR = 1.637) could be regarded as independent risk factors for overall survival of HCC patients (**Table 2**). Moreover, tumor TNM stage (P < 0.001, HR = 2.188) and high HNRNPC level (P = 0.002, HR = 1.883) could independently predict disease-free survival in HCC patients (**Table 2**).

**Table 2:** Univariate and multivariate analysis for survival of 147 HCC patients.

Clinical features	Univariate		Multivariate	
	HR (95% CI)	P-value	HR (95% CI)	P-value
Overall survival:				
Gender (female vs male)	0.938 (0.498–1.767)	0.843		
Age (≥55 y vs <55 y)	1.068 (0.698–1.636)	0.761		
HBV infection (negative vs positive)	0.717 (0.389–1.321)	0.286		
Liver cirrhosis (absent vs present)	0.675 (0.425–1.072)	0.095		
Child–Pugh (B vs A)	1.295 (0.761–2.205)	0.341		
Tumor number (2–3 vs 1)	1.392 (0.784–2.470)	0.259		
Tumor size (≥5 cm vs <5 cm)	2.501 (1.598–3.915)	**<0.001**	1.957 (1.224–3.135)	**0.005**
TNM stage (II–III vs I)	2.515 (1.634–3.871)	**<0.001**	1.786 (1.124–2.841)	**0.014**
Tumor differentiation (III–IV vs I–II)	1.017 (0.629–1.643)	0.946		
AFP (≥400 vs <400)	0.763 (0.496–1.173)	0.218		
HNRNPC (high vs low)	2.117 (1.349–3.322)	**0.001**	1.637 (1.025–2.611)	**0.039**
Disease-free survival:				
	
Gender (female vs male)	0.876 (0.501–1.536)	0.645		
Age (≥55 y vs <55 y)	1.090 (0.753–1.577)	0.649		
HBV infection (negative vs positive)	0.736 (0.439–1.233)	0.244		
Liver cirrhosis (absent vs present)	0.693 (0.466–1.028)	0.068		
Child–Pugh (B vs A)	1.170 (0.728–1.881)	0.516		
Tumor number (2–3 vs 1)	1.959 (1.184–3.241)	**0.009**	1.093 (0.620–1.927)	0.760
Tumor size (≥5 cm vs <5 cm)	1.830 (1.257–2.662)	**0.002**	1.348 (0.905–2.004)	0.142
TNM stage (II–III vs I)	2.572 (1.750–3.779)	**<0.001**	2.188 (1.471–3.257)	**<0.001**
Tumor differentiation (III–IV vs I–II)	1.099 (0.729–1.658)	0.652		
AFP (≥400 vs <400)	1.387 (0.954–2.012)	0.086		
HNRNPC (high vs low)	2.249 (1.525–3.317)	**<0.001**	1.883 (1.261–2.817)	**0.002**

HNRNPC, heterogeneous nuclear ribonucleoprotein C; HBV, hepatitis B virus; AFP, alpha fetoprotein. The bold values present statistical significance.

### Knockdown of HNRNPC Suppresses HCC Proliferation, Invasion, and Migration *In Vitro*

**Figure 2:**
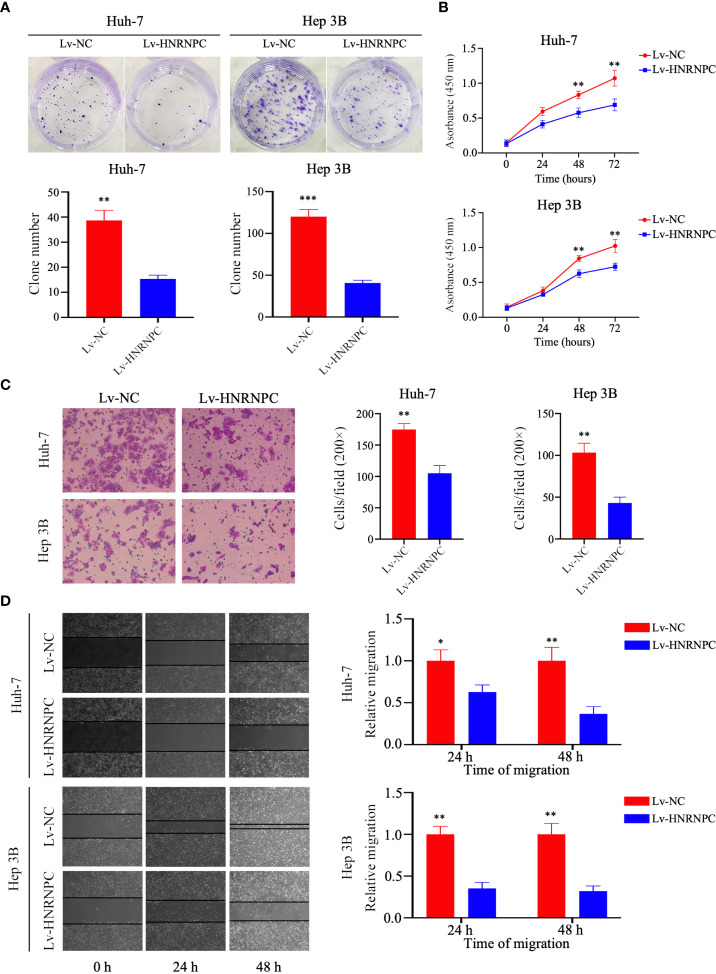
Knockdown of HNRNPC suppressed proliferation, invasion, and migration of HCC cells *in vitro*. **(A)** Colony formation assay showing the impact of HNRNPC knockdown on HCC cell proliferation. **(B)** CCK-8 test showing the effects of HNRNPC downregulation on HCC cell viability. **(C)** Transwell assay showing the influence of HNRNPC inhibition on HCC cell invasion. **(D)** Scratch wound test showing the influence of HNRNPC suppression on HCC cell migration. All experiments were independently repeated three times, and the results were showed as mean ± standard deviation (SD). ^*^P < 0.05, ^**^P < 0.01, ^***^P < 0.001.

To observe the impact of HNRNPC on HCC proliferation, invasion, and migration, Lv-NC and Lv-HNRNPC lentiviruses were used to infect the Huh-7 and Hep 3B cell lines. After selection by puromycin, the infection efficiency of Huh-7 and Hep 3B cells was >90% (**Supplementary Figure 1A**), and lower HNRNPC expression was detected in Lv-HNRNPC Huh-7 and Hep 3B cells *via* western blotting and q-RT-PCR (**Supplementary Figures 1B, C**). CCK-8 and colony formation assays were used to analyze cell proliferation *in vitro*. The colony formation assay revealed that HNRNPC knockdown significantly inhibited proliferation of Huh-7 and Hep 3B cells (**Figure 2A**). In the CCK-8 test, the viability of Huh-7 and Hep 3B cells was also significantly suppressed by silencing of HNRNPC (**Figure 2B**). A transwell assay was performed to observe invasion; the results showed that inhibition of HNRNPC impaired the invasion activity of Huh-7 and Hep 3B cells (**Figure 2C**). The scratch wound assay showed decreased migration ability of Huh-7 and Hep 3B cells in the Lv-HNRNPC-group (**Figure 2D**).

### Inhibition of HNRNPC Downregulates the Activity of the MAPK Signaling Pathway, Blocks Tumors at G0/G1 Phase, and Suppresses EMT Process *In Vitro*

**Figure 3:**
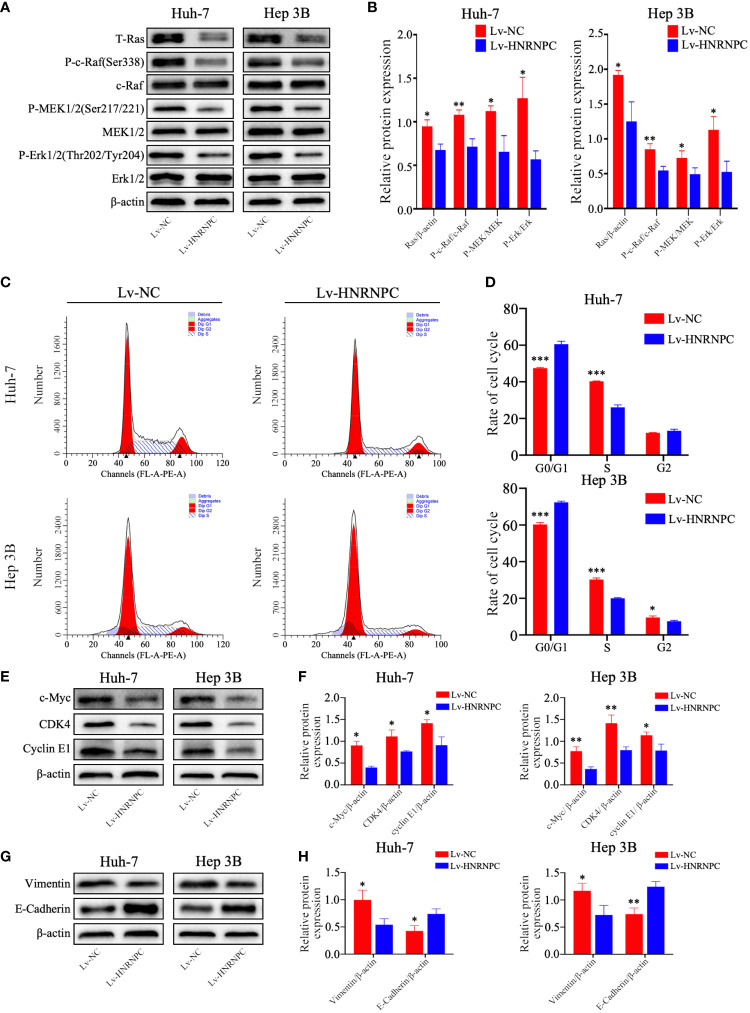
HNRNPC suppression inhibited the Ras/MAPK signaling pathway, leading to arrest of more cells at S phase and blocking of EMT *in vitro*. **(A, B)** Western blot to detect activation of the Ras/MAPK signaling pathway in Lv-NC Huh-7, Lv-HNRNPC Huh-7, Lv-NC Hep 3B, and Lv-HNRNPC Hep 3B cells. **(C, D)** Cell cycle analysis for the various cell groups mentioned above. **(E, F)** Western blot to detect c-Myc, CDK4, and cyclin E1 levels in the various cell groups. **(G, H)** Western blot to analyze Vimentin and E-Cadherin expression in various cell groups. Each test was performed independently in triplicate, and the results were presented as mean ± standard deviation (SD). ^*^P < 0.05, ^**^P < 0.01, ^***^P < 0.001.

To study the potential molecular mechanisms by which HNRNPC suppressed tumor growth, migration and invasion, we selected 3517 genes that were significantly correlated with HNRNPC (Pearson correlation coefficient ≥ 0.5) from the UALCAN database; these genes were subjected to KEGG analysis using WebGestalt (2013). Among the enriched KEGG pathways, “Cell cycle” and “Pathways in cancer” were highly related to tumor growth (**Supplementary Figure 1D**), and “Pathways in cancer” mainly involved the MAPK and AKT signaling pathways (**Supplementary Figure 1E**). Subsequently, the activity of the MAPK signaling pathway was detected by western blotting. The results showed that HNRNPC knockdown statistically decreased the expression of Ras and the relative levels of p-Raf, p-MEK1/2, and p-Erk1/2 in Huh-7 and Hep 3B cells (**Figures 3A, B**). As the MAPK pathway activates a series of downstream genes to regulate the cell cycle, thereby regulating cell proliferation, we used flow cytometry for cell cycle analysis. The results showed that HNRNPC downregulation blocked more cells at G0/G1 phase and reduced the proportion of S-phase cells among both Huh-7 and Hep 3B cells (**Figures 3C, D**). CDK4, cyclin E1, and c-myc are well known as important molecules that positively regulate G1-S transition. In the present study, silencing of HNRNPC markedly downregulated CDK4, cyclin E1, and c-myc protein levels, as confirmed by western blotting (**Figures 3E, F**). Ras/MAPK signaling pathway not only positively regulate G1-S transition to promote tumor proliferation, but also accelerate EMT process to facilitate tumor migration and invasion. We also detected the marker of EMT by western blotting, our results indicated that HNRNPC silencing decreased Vimentin level, and upregulated E-cadherin expression statistically (**Figures 3G, H**).

### HNRNPC Silencing Inhibits Tumor Growth *In Vivo*

**Figure 4:**
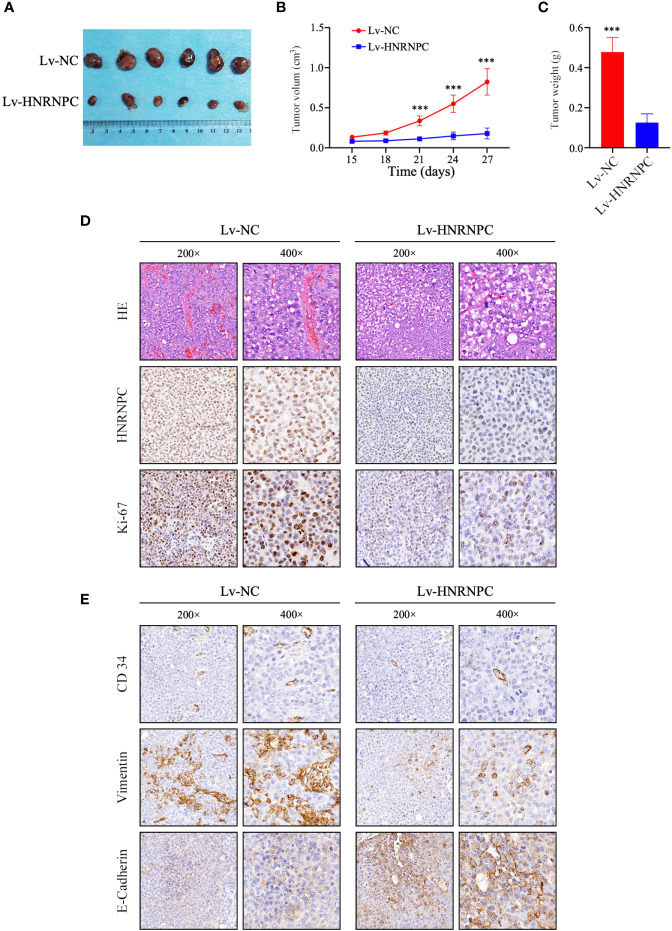
HNRNPC knockdown blocked HCC cell proliferation *in vivo*. **(A)** Huh-7-cell xenograft tumors in Lv-HNRNPC and Lv-NC groups. **(B, C)** t-test analysis showing tumor weights and sizes in Lv-HNRNPC and Lv-NC Huh-7 cell groups. **(D)** H&E staining to confirm xenograft tumors (upper panel); IHC to detect HNRNPC levels in xenograft tumors (middle panel); proliferation of xenograft tumors as determined by Ki-67 staining (nether panel). **(E)** IHC staining to analyze CD34 (upper panel), Vimentin (middle panel), and E-Cadherin (nether panel) expression in xenograft tumors. The tumor size and weight were demonstrated as mean ± standard deviation (SD). ***P < 0.001.

To explore the impact of HNRNPC on tumor growth *in vivo*, Lv-NC and Lv-HNRNPC Huh-7 cells were implanted in nude mice subcutaneously. The tumors of the Lv-HNRNPC group showed obvious reductions in both size and weight compared with those of the Lv-NC group (**Figures 4A–C**). Subsequently, hematoxylin eosin (H&E) and IHC staining were performed to confirm the tumor tissues and HNRNPC knockdown efficiency, respectively (**Figure 4D**). In addition, Ki-67 was used to observe the proliferation of tumor cells *via* IHC; the Ki-67 staining intensity was stronger in the Lv-NC group than in the Lv-HNRNPC group (**Figure 4D**). Tumor migration and invasion are highly correlated with angiogenesis and EMT process, so Vimentin, E-cadherin, and CD34 were used to evaluate the ability of tumor invasion and migration by IHC. The results showed that Vimentin and CD34 staining intensity were weaker, and E-cadherin staining intensity was stronger in the Lv-HNRNPC group than in Lv-NC group (**Figure 4E**).

### Ras Agonist Partly Recovers Growth, Migration, and Invasion Ability in Lv-HNRNPC Group

**Figure 5:**
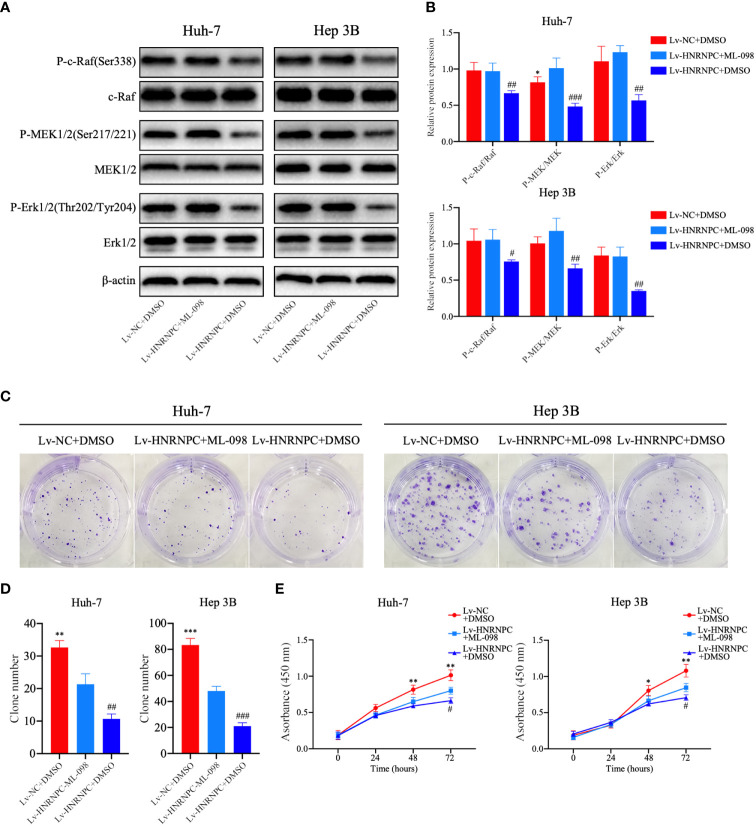
After treatment with ML-098 (20 nmol/l), MAPK signaling pathway activation was recovered completely in Lv-HNRNPC-group cells, and proliferation ability was partially reversed. **(A, B)** Western blot to detect recovery of activation of the MAPK signaling pathway in Lv-HNRNPC groups after treatment with ML-098. **(C, D)** Colony formation assay showing the reversed efficiency of ML-098 in Lv-HNRNPC group cell proliferation. **(E)** CCK-8 test showing the rescue by ML-098 of cell viability in the Lv-HNRNPC group. The tests mentioned above were operated three times respectively, and the results were reported as mean ± standard deviation (SD). ^*^P < 0.05, ^**^P < 0.01, ^***^P < 0.001, for Lv-NC+DMSO group compared with Lv-HNRNPC+ML-098 group; ^#^P < 0.05, ^##^P < 0.01, ^###^P < 0.001, for Lv-HNRNPC+DMSO group compared with Lv-HNRNPC+ML-098 group.

**Figure 6:**
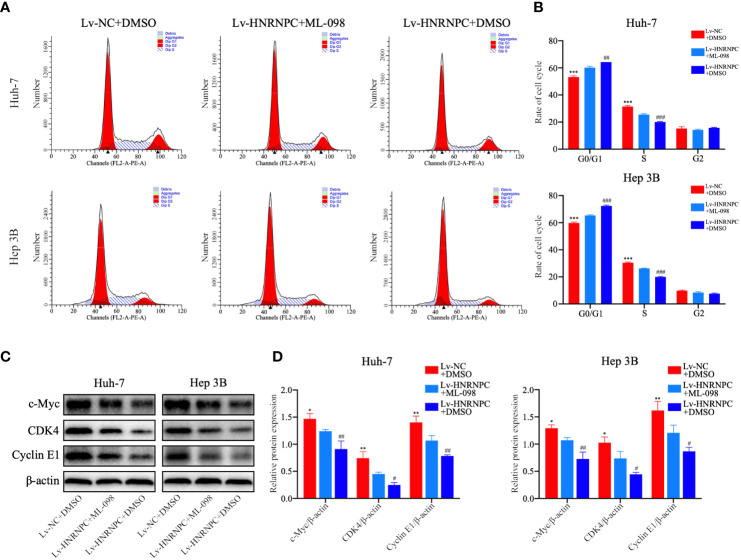
ML-098 partly reversed the reduction in numbers of S phase cells in the Lv-HNRNPC group. **(A, B)** Cell cycle analysis to detect the recovery of numbers of S phase cells in the Lv-HNRNPC groups after treatment with ML-098. **(C, D)** Western blot to detect the recovery of c-Myc, CDK4, and cyclin E1 in Lv-HNRNPC cell groups after treatment with ML-098. All tests were repeated independently in triplicate, and the results were showed as mean ± standard deviation (SD). ^*^P < 0.05, ^**^P < 0.01, ^***^P < 0.001, for Lv-NC+DMSO group compared with Lv-HNRNPC+ML-098 group; ^#^P < 0.05, ^##^P < 0.01, ^###^P < 0.001, for Lv-HNRNPC+DMSO group compared with Lv-HNRNPC+ML-098 group.

**Figure 7:**
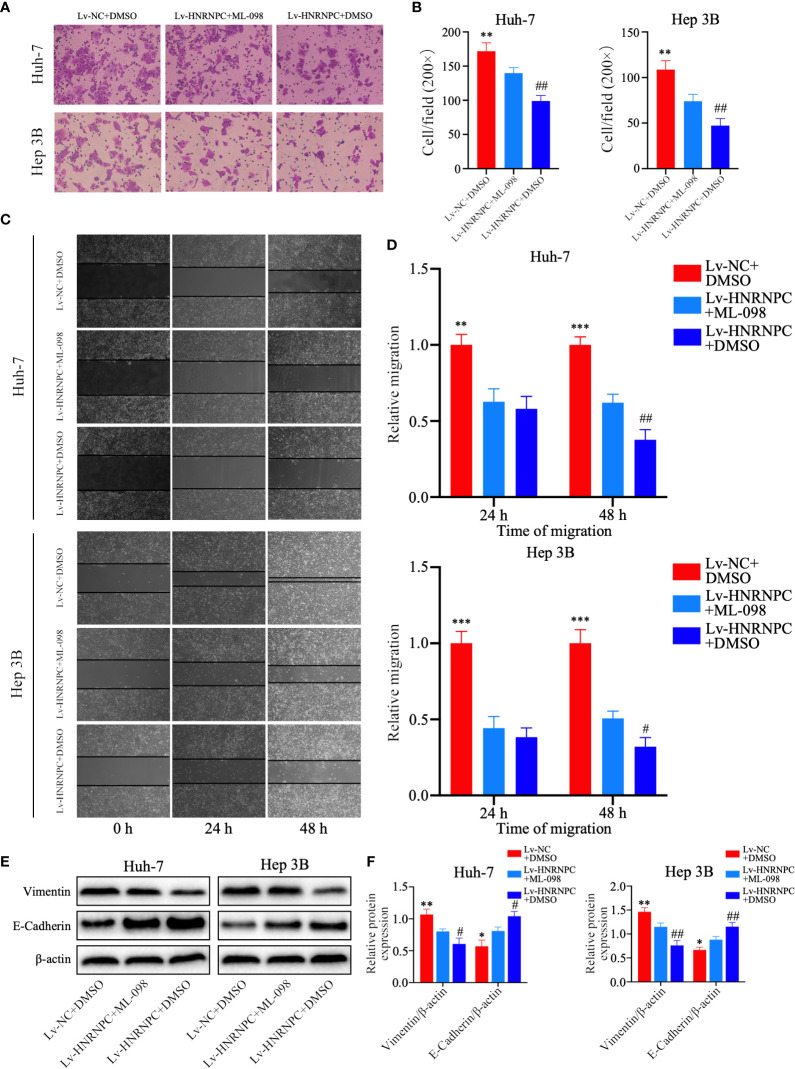
ML-098 recovered the invasion and migration abilities of Lv-HNRNPC group cells in part. **(A, B)** Transwell assay demonstrating the recovered efficiency of ML-098 in Lv-HNRNPC group cell invasion. **(C, D)** Scratch wound test showing the rescue by ML-098 of cell migration in the Lv-HNRNPC group. **(E, F)** Western blot to detect the recovery of EMT process in Lv-HNRNPC cell groups after treatment with ML-098. Each test was repeated independently in triplicate, and the results were demonstrated as mean ± standard deviation (SD). ^*^P < 0.05, ^**^P < 0.01, ^***^P < 0.001, for Lv-NC+DMSO group compared with Lv-HNRNPC+ML-098 group; ^#^P < 0.05, ^##^P < 0.01, for Lv-HNRNPC+DMSO group compared with Lv-HNRNPC+ML-098 group.

CDK4, cyclin E1, and c-myc are classical downstream genes of the MAPK signaling pathway. To confirm the upstream and downstream relationships between the MAPK signaling pathway and these cell cycle-related molecules, a Ras agonist (ML-098) was used to treat cells in the Lv-HNRNPC group. Details of this process are given in the Materials and Methods section. First, we performed western blotting to detect the recovery of MAPK pathway activity. After treatment with ML-098 for 48 h, the activity of the MAPK pathway was reversed completely in the Lv-HNRNPC group (**Figures 5A, B**). Moreover, colony formation and CCK-8 assays revealed that the proliferation ability of cells in the Lv-HNRNPC group was partly rescued by ML-098 (**Figures 5C–E**). Furthermore, the cell cycle assay showed that ML-098 treatment could partially reverse the G0/G1 arrest caused by HNRNPC knockdown in both Huh-7 and Hep 3B cells (**Figures 6A, B**). Finally, using western blotting, we showed that the effects of HNRNPC inhibition on CDK4, cyclin E1, and c-myc levels in HCC cells were reversed by ML-098 to some extent (**Figures 6C, D**). On the other hand, Ras/MAPK signaling pathway take part in EMT process. In present study, cell invasion and scratch wound assays showed that the invasion (**Figures 7A, B**) and migration (**Figures 7C, D**) abilities of the Lv-HNRNPC group cells were recovered by ML-098 partly. Furthermore, the western blotting results demonstrated that after treatment with ML-098, the EMT process inhibition of cells in the Lv-HNRNPC group was reversed in part too (**Figures 7E, F**). Taken together, these results indicate that HNRNPC inhibition not only arrests HCC cells in G0/G1 phase to inhibit tumor proliferation, but also suppresses EMT process to block invasion and migration of HCC cells in part *via* the Ras/Raf/MEk/Erk signaling pathway.

## Discussion

With progress in medical treatment, molecular therapies are increasingly available for advanced HCC patients (51–53); however, the prognosis of these patients remains poor (1). This prompted us to explore novel pathogenetic mechanisms and therapeutic targets for HCC. According to present evidence, as a process fundamental to cancer (54), aberrant alternative splicing occurs more frequently in various malignant tumors, including renal cancer (55), lung cancer, and HCC (56); this makes it possible for cells with phenotypic variability to adapt to unfamiliar microenvironments, treatments, and immune responses (57). HNRNPs, which include HNRNP A2/B1, HNRNP L, HNRNP H, and HNRNPC, are primary factors for alternative splicing. HNRNPC is believed to be the founder of the HNRNP family (58), that is, it seems to be at the core of regulating alternative splicing. The relationships of HNRNP L (59), HNRNP A2/B1 (60), and HNRNP H (61) with HCC have been reported; however, any association of HNRNPC with HCC remained unknown.

Using two-dimensional difference electrophoresis, Sun et al. found that HNRNPC protein expression was higher in HCC tumor tissues than normal liver tissues (62). However, they did not further investigate any correlation of HNRNPC protein expression with prognosis or clinicopathologic characteristics in HCC. In the present study, we showed that HNRNPC protein levels were statistically higher in HCC tumor tissues than in para-tumor tissues by western blotting and IHC. Moreover, Kaplan–Meier analysis and Cox proportional hazards regression models were used to perform survival analysis; this demonstrated that a high HNRNPC protein level was an independent risk factor that could serve as a biomarker predicting poor overall survival and disease-free survival in HCC patients. By functional experiments, we showed that HNRNPC knockdown significantly inhibited proliferation, migration, and invasion of HCC cells *in vitro*. Finally, the xenograft assay showed that suppressing HNRNPC significantly restrained tumor growth *in vivo*.

HNRNPC has an important role in RNA combination and alternative splicing. Fischl et al. reported that HNRNPC regulates alternative cleavage and polyadenylation (APA) profiles in colon cancer, and by coding region APA, HNRNPC mainly affected MTHFD1L protein levels, which are strongly linked to tumor progression (24). In addition, Wu et al. extended the function of HNRNPC to alternative splicing in breast cancer: by controlling endogenous double-stranded RNA, HNRNPC regulated the activation of the IFNβ signaling pathway to affect the progression of breast cancer (27). In the present study, we performed bioinformatic analysis of HNRNPC-correlated genes, which brought the Ras/Raf/MEK/Erk and AKT signaling pathways to our attention. HNRNPC has previously been reported to cause AKT phosphorylation in ovarian cancer (32). We used western blotting to detect the activation of the Ras/Raf/MEK/Erk signaling pathway; the results indicated that HNRNPC knockdown significantly inhibited the activation of the MAPK signaling pathway. Ras mutation has a very important role in many cancers (63–65), and HCC is no exception (66, 67). Both H-ras and K-ras have respective splice variants (68, 69), which could be regarded as Ras mutations at the mRNA level. Barbier et al. reported that H-ras splice variants including premature translation termination codons were selectively degraded by the nonsense-mediated mRNA decay pathway to regulate Ras expression (69). However, the mechanism by which Ras downregulation was caused by HNRNPC inhibition was not investigated in our study; this will be explored in the future. This novel means of Ras mutation may provide a new approach to HCC therapy.

The MAPK signaling pathway regulates the majority of cell functions, in particular, cell proliferation, apoptosis, and metabolism (70–72). After being activated, the Ras cascade phosphorylates Raf, Mek, and Erk to regulate cell proliferation and apoptosis by controlling G1-S transition (73, 74). In the present study, we prove that inhibition of the MAPK signaling pathway by HNRNPC suppression causes the arrest of more HCC cells at G0/G1 phase. CDK4 (75), cyclin E1 (76), and c-myc (77) play important parts in regulating G1-S transition. As G1-S transition overactivation occurs in the majority of malignant tumors, inhibitors of CDK4 and cyclin E1 have emerged as candidate drugs for tumor treatment (78, 79). In our study, we showed that HNRNPC knockdown statistically inhibited CDK4, cyclin E1, and c-myc expression. Subsequently, rescue experiments revealed that activation of the MAPK signaling pathway could partly recover CDK4, cyclin E1, and c-myc expression, demonstrating that knockdown of HNRNPC induced G0/G1 arrest in part *via* the MAPK signaling pathway. Epithelial-mesenchymal transition is an essential factor to initiate tumor migration and invasion (80). In recent years, more and more research indicated that Ras/MAPK signaling pathway play an important role in EMT process (43, 81, 82). In the present study, we proved that HNRNPC inhibition suppressed the EMT process of HCC cells. Additionally, after treatment with ML-098, the EMT process of HCC cells in Lv-HNRNPC group was recovered in part, which support the above point again. Ras is the second mutated gene driver in many malignant tumors (83), and Ras/MAPK have been shown to be activated in 50–100% of HCC patients (84). MAPK-associated inhibitors have been used to treat HCC (85), but very few HCC patients benefited from this treatment (86), largely owing to the compensatory activation of other Ras-related pathways such as the AKT (87) and IGF/FGF (88) signaling pathways. Thus, exploration of the mechanisms of Ras mutation may provide a novel approach for HCC therapy. In our study, we reported that HNRNPC knockdown inhibited Ras/MAPK activation to block proliferation of HCC cells, which may represent a new mutation mechanism for Ras. However, the present study did not consider how HNRNPC regulates Ras; this will be explored in our future studies to determine the mechanisms of novel Ras mutations and offer a new approach for HCC therapy.

## Data Availability Statement

The original contributions presented in the study are included in the article/**Supplementary Material**. Further inquiries can be directed to the corresponding authors.

## Ethics Statement

The studies involving human participants were reviewed and approved by Ethics Committee at the Second Affiliated Hospital of Chongqing Medical University. The patients/participants provided their written informed consent to participate in this study. The animal study was reviewed and approved by Chongqing Municipal Committee of Science Technology.

## Author Contributions

The study was conceived and designed by JG. G-CZ, JH, and DC wrote the manuscript and performed the experiments. The statistical analysis was carried out by ZZ. All authors contributed to the article and approved the submitted version.

## Conflict of Interest

The authors declare that the research was conducted in the absence of any commercial or financial relationships that could be construed as a potential conflict of interest.

## Supplementary Material

Supplementary Figure 1 Knockdown efficiency of HNRNPC in Huh-7 and Hep 3B cells, and KEGG analysis. **(A)** EGFP expression showing the infective efficiency of the lentivirus. **(B)** Western blot to detect knockdown efficiency of HNRNPC in Huh-7 and Hep 3B cells. **(C)** q-RT-PCR to detect knockdown efficiency of HNRNPC in Huh-7 and Hep 3B cells. **(D, E)** KEGG analysis of 3517 HNRNPC-correlated genes by WebGestalt 2013. ***P<0.001.

## Abbreviations

HCC, hepatocellular carcinoma; HNRNPC, heterogeneous nuclear ribonucleoprotein C; IHC, immunohistochemical; MAPK, mitogen-activated protein kinase; AFP, alpha fetoprotein; EMT, epithelial-mesenchymal transition; HBV, hepatitis B virus; Lv-NC, hepatocellular carcinoma cell lines infected with negative control lentiviruses; Lv-HNRNPC, hepatocellular carcinoma cell lines infected with targeted HNRNPC lentiviruses; EMT, epithelial-mesenchymal transition.
