# Supplementary material for: Suppression of Heterogeneous Nuclear Ribonucleoprotein C Inhibit Hepatocellular Carcinoma Proliferation, Migration, and Invasion via Ras/MAPK Signaling Pathway
Source: Front Oncol. 2021 Apr 16;11:659676. doi: 10.3389/fonc.2021.659676 (PMC8087488; doi:10.3389/fonc.2021.659676)
Supplement: Supplementary Figure 1 — Knockdown efficiency of HNRNPC in Huh-7 and Hep 3B cells, and KEGG analysis. (A) EGFP expression showing the infective efficiency of the lentivirus. (B) Western blot to detect knockdown efficiency of HNRNPC in Huh-7 and Hep 3B cells. (C) q-RT-PCR to detect knockdown efficiency of HNRNPC in Huh-7 and Hep 3B cells. (D, E) KEGG analysis of 3517 HNRNPC-correlated genes by WebGestalt 2013. ***P<0.001. [file DataSheet_1.docx]

Supplementary Material

## Supplementary Figures

**Figure 1**

**
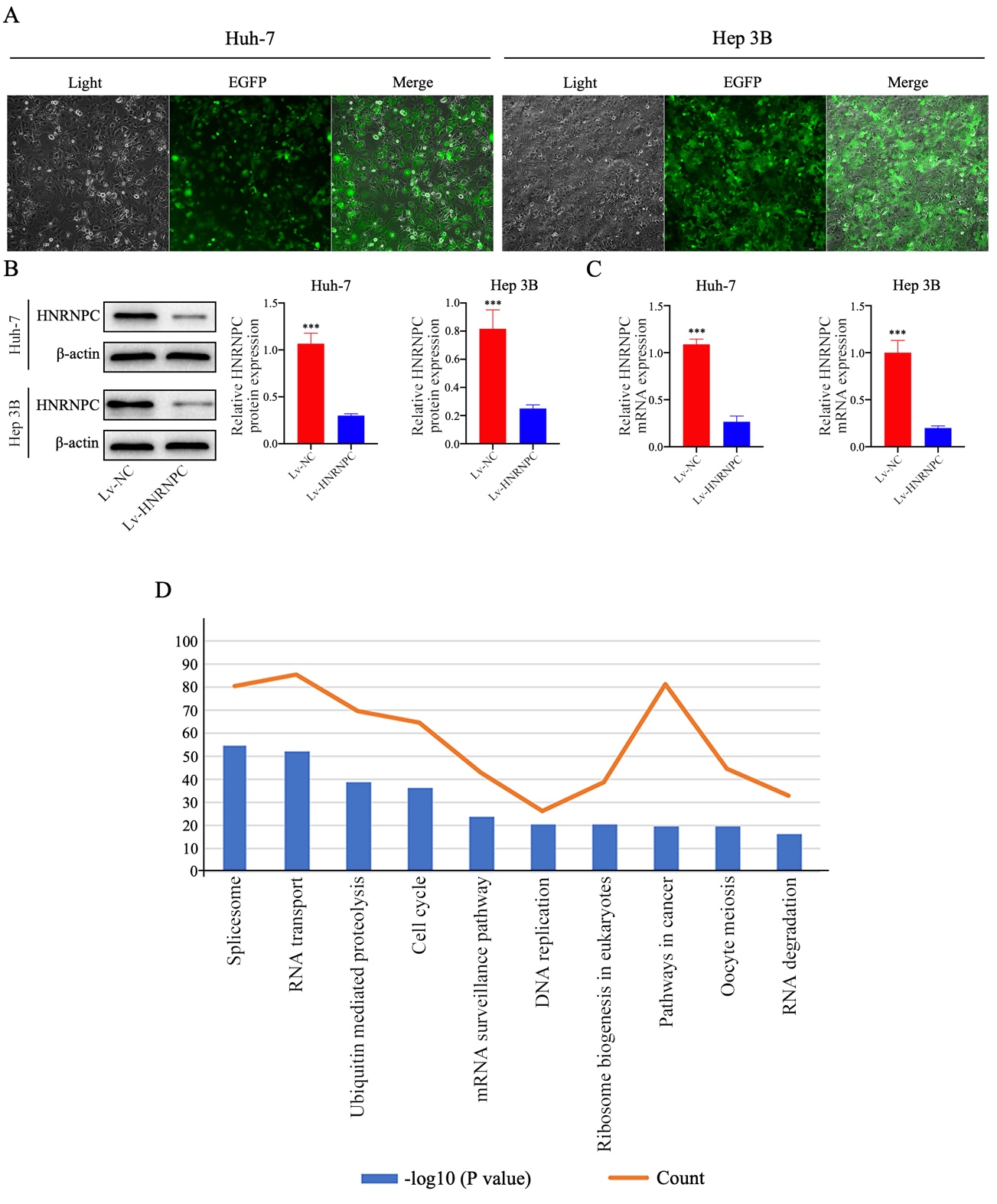
**

**Supplementary Figure 1.** Knockdown efficiency of HNRNPC in Huh-7 and Hep 3B cells, and KEGG analysis. **(A)** EGFP expression showing the infective efficiency of the lentivirus. **(B)** Western blot to detect knockdown efficiency of HNRNPC in Huh-7 and Hep 3B cells. **(C)** q-RT-PCR to detect knockdown efficiency of HNRNPC in Huh-7 and Hep 3B cells. **(D)** KEGG analysis of 3517 HNRNPC-correlated genes by WebGestalt 2013. The western blotting and q-PCR assays were performed three times in independence, and the final results were presented as mean ± standard deviation (SD). ^***^P<0.001.

**Figure 2**


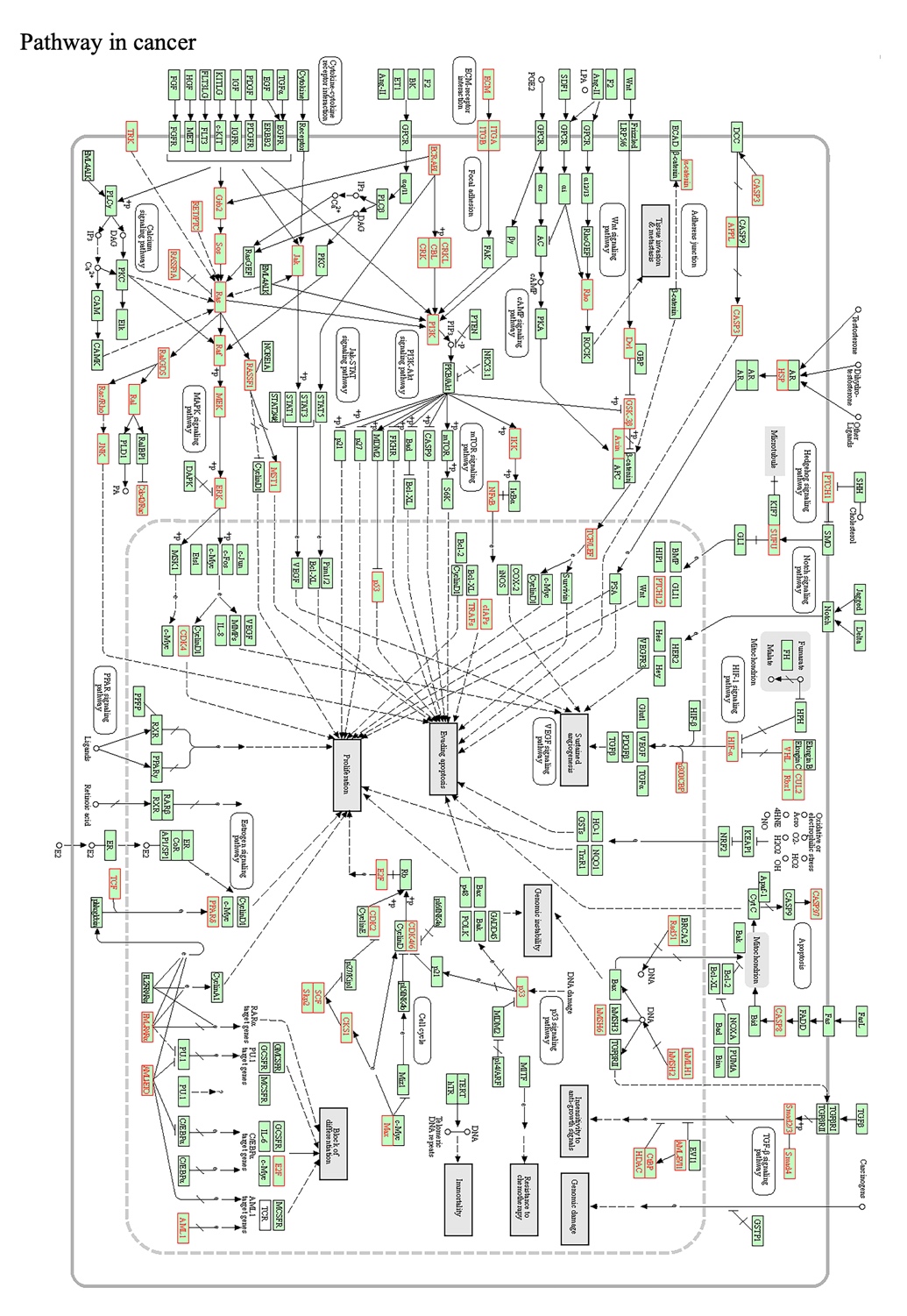


**Supplementary Figure 2.** The content of pathway in cancer from WebGestalt 2013 analysis.
